# Creatine supplementation with specific view to exercise/sports performance: an update

**DOI:** 10.1186/1550-2783-9-33

**Published:** 2012-07-20

**Authors:** Robert Cooper, Fernando Naclerio, Judith Allgrove, Alfonso Jimenez

**Affiliations:** 1 Centre for Sports Science and Human Performance, School of Science, University of Greenwich at Medway, Central Avenue, Chatham Maritime, Kent, ME4 4TB, United Kingdom; 2 Institute of Sport, Exercise and Active Living, ISEAL, Victoria University, Melbourne, Australia

## Abstract

Creatine is one of the most popular and widely researched natural supplements. The majority of studies have focused on the effects of creatine monohydrate on performance and health; however, many other forms of creatine exist and are commercially available in the sports nutrition/supplement market. Regardless of the form, supplementation with creatine has regularly shown to increase strength, fat free mass, and muscle morphology with concurrent heavy resistance training more than resistance training alone. Creatine may be of benefit in other modes of exercise such as high-intensity sprints or endurance training. However, it appears that the effects of creatine diminish as the length of time spent exercising increases. Even though not all individuals respond similarly to creatine supplementation, it is generally accepted that its supplementation increases creatine storage and promotes a faster regeneration of adenosine triphosphate between high intensity exercises. These improved outcomes will increase performance and promote greater training adaptations. More recent research suggests that creatine supplementation in amounts of 0.1 g/kg of body weight combined with resistance training improves training adaptations at a cellular and sub-cellular level. Finally, although presently ingesting creatine as an oral supplement is considered safe and ethical, the perception of safety cannot be guaranteed, especially when administered for long period of time to different populations (athletes, sedentary, patient, active, young or elderly).

## Introduction

Creatine is produced endogenously at an amount of about 1 g/d. Synthesis predominately occurs in the liver, kidneys, and to a lesser extent in the pancreas. The remainder of the creatine available to the body is obtained through the diet at about 1 g/d for an omnivorous diet. 95% of the bodies creatine stores are found in the skeletal muscle and the remaining 5% is distributed in the brain, liver, kidney, and testes [1]. As creatine is predominately present in the diet from meats, vegetarians have lower resting creatine concentrations [2].

Creatine is used and researched in a clinical setting to investigate various pathologies or disorders such as myopathies [3,4] and is also used as an ergogenic aid for improving health and sports performance in athletes [5]. As an oral supplement, the most widely used and researched form is creatine monohydrate (CM). When orally ingested, CM has shown to improve exercise performance and increase fat free mass [5-9].

There is a great amount of research published on creatine supplementation; protocols of administration, forms of creatine, as well as potential side effects. Despite this, the mechanisms by which creatine acts in the human body to improve physical and cognitive performance are still not clear. The main objectives of this review are to analyze the more recent findings on the effects and mechanisms of creatine supplementation in sports and health. As a secondary purpose, we will analyze the most recommended protocols of ingestion and its potential side effects.

## Creatine metabolism

The majority of creatine in the human body is in two forms, either the phosphorylated form making up 60% of the stores or in the free form which makes up 40% of the stores. The average 70 kg young male has a creatine pool of around 120-140 g which varies between individuals [10,11] depending on the skeletal muscle fiber type [1] and quantity of muscle mass [11]. The endogenous production and dietary intake matches the rate of creatinine production from the degradation of phosphocreatine and creatine at 2.6% and 1.1%/d respectively. In general, oral creatine supplementation leads to an increase of creatine levels within the body. Creatine can be cleared from the blood by saturation into various organs and cells or by renal filtration [1].

Three amino acids (glycine, arginine and methionine) and three enzymes (L-arginine:glycine amidinotransferase, guanidinoacetate methyltransferase and methionine adenosyltransferase) are required for creatine synthesis. The impact creatine synthesis has on glycine metabolism in adults is low, however the demand is more appreciable on the metabolism of arginine and methionine [11].

Creatine ingested through supplementation is transported into the cells exclusively by CreaT1. However, there is another creatine transporter Crea T2, which is primarily active and present in the testes [12]. Creatine uptake is regulated by various mechanisms, namely phosphorylation and glycosylation as well as extracellular and intracellular levels of creatine. Crea T1 has shown to be highly sensitive to the extracellular and intracellular levels being specifically activated when total creatine content inside the cell decreases [12]. It has also been observed that in addition to cytosolic creatine, the existence of a mitochondrial isoform of Crea T1 allows creatine to be transported into the mitochondria. Indicating another intra-mitochondrial pool of creatine, which seems to play an essential role in the phosphate-transport system from the mitochondria to the cytosol [13]. Myopathy patients have demonstrated reduced levels of total creatine and phosphocreatine as well as lower levels of CreaT1 protein, which is thought to be a major contributor to these decreased levels [14].

## Documented effects of creatine supplementation on physical performance

The majority of studies focusing on creatine supplementation report an increase in the body’s’ creatine pool [15-17]. There is a positive relationship between muscle creatine uptake and exercise performance [17]. Volek et al [18] observed a significant increase in strength performance after 12 weeks creatine supplementation with a concurrent periodized heavy resistance training protocol. The creatine supplementation protocol consisted of a weeklong loading period of 25 g/d followed by a 5 g maintenance dose for the remainder of the training. These positive effects were attributed to an increased total creatine pool resulting in more rapid adenosine triphosphate (ATP) regeneration between resistance training sets allowing athletes to maintain a higher training intensity and improve the quality of the workouts along the entire training period.

It is regularly reported that creatine supplementation, when combined with heavy resistance training leads to enhanced physical performance, fat free mass, and muscle morphology [18-22]. A 2003 meta analysis [8] showed individuals ingesting creatine, combined with resistance training, obtain on average +8% and +14% more performance on maximum (1RM) or endurance strength (maximal repetitions at a given percent of 1RM) respectively than the placebo groups. However, contradicting studies have reported no effects of creatine supplementation on strength performance. Jakobi et al [23] found no effects of a short term creatine loading protocol upon isometric elbow flexion force, muscle activation, and recovery process. However, this study did not clearly state if creatine supplementation was administered concurrent with resistance training. Bemben et al [24] have shown no additional benefits of creatine alone or combined with whey protein for improving strength and muscle mass after a progressive 14 weeks (3 days per week) resistance training program in older men. These conflicting results can be explained by the possibility that the supplemented groups were formed by a greater amount of non-responders or even because creatine supplementation was administered on the training days only (3 times a week). This strategy has not been adequately tested as effective in middle aged and older men for maintaining post loading elevated creatine stores [5].

A quantitative, comprehensive scientific summary and view of knowledge up to 2007 on the effects of creatine supplementation in athletes and active people was published in a 100 citation review position paper by the *International Society of Sports Nutrition*[5]. More recent literature has provided greater insight into the anabolic/performance enhancing mechanisms of creatine supplementation [15,25] suggesting that these effects may be due to satellite cell proliferation, myogenic transcription factors and insulin-like growth factor-1 signalling [16]. Saremi et al [26] reported a change in myogenic transcription factors when creatine supplementation and resistance training are combined in young healthy males. It was found that serum levels of myostatin, a muscle growth inhibitor, were decreased in the creatine group.

Collectively, in spite of a few controversial results, it seems that creatine supplementation combined with resistance training would amplify performance enhancement on maximum and endurance strength as well muscle hypertrophy.

## Effects of creatine supplementation on predominantly anaerobic exercise

Creatine has demonstrated neuromuscular performance enhancing properties on short duration, predominantly anaerobic, intermittent exercises. Bazzucch et al [27] observed enhanced neuromuscular function of the elbow flexors in both electrically induced and voluntary contractions but not on endurance performance after 4 loading doses of 5 g creatine plus 15 g maltodextrin for 5/d in young, moderately trained men. Creatine supplementation may facilitate the reuptake of Ca^2+^ into the sacroplasmic reticulum by the action of the Ca^2+^ adenosine triphosphatase pump, which could enable force to be produced more rapidly through the faster detachment of the actomyosin bridges.

A previous meta-analysis [28] reported an overall creatine supplementation effect size (ES) of 0.24 ± 0.02 for activities lasting ≤30 s. (primarily using the ATP- phosphocreatine energy system). For this short high-intensity exercise, creatine supplementation resulted in a 7.5 ± 0.7% increase from base line which was greater than the 4.3 ± 0.6% improvement observed for placebo groups. When looking at the individual selected measures for anaerobic performance the greatest effect of creatine supplementation was observed on the number of repetitions which showed an ES of 0.64 ± 0.18. Furthermore, an increase from base line of 45.4 ± 7.2% compared to 22.9 ± 7.3% for the placebo group was observed. The second greatest ES was on the weight lifted at 0.51 ± 0.16 with an increase from base line of 13.4 ± 2.7% for the placebo group and 24.7 ± 3.9% for the creatine group. Other measures improved by creatine with a mean ES greater than 0 were for the amount of work accomplished, weight lifted, time, force production, cycle ergometer revolutions/min and power. The possible effect of creatine supplementation on multiple high intensity short duration bouts (<30 s) have shown an ES not statistically significant from 0. This would indicate that creatine supplementation might be useful to attenuate fatigue symptoms over multiple bouts of high-intensity, short duration exercise. The ES of creatine on anaerobic endurance exercise (>30 – 150s), primarily using the anaerobic glycolysis energy system, was 0.19 ± 0.05 with an improvement from baseline of 4.9 ± 1.5 % for creatine and -2.0 ± 0.6% for the placebo. The specific aspects of anaerobic endurance performance improved by creatine supplementation were work and power, both of which had a mean ES greater than 0. From the findings of this previous meta-analysis [28] it would appear that creatine supplementation has the most pronounced effect on short duration (<30s) high intensity intermittent exercises.

## Effects of creatine supplementation on skeletal muscle hypertrophy

Cribb et al (2007) [29] observed greater improvements on 1RM, lean body mass, fiber cross sectional area and contractile protein in trained young males when resistance training was combined with a multi-nutrient supplement containing 0.1 g/kg/d of creatine, 1.5 g/kg/d of protein and carbohydrate compared with protein alone or a protein carbohydrate supplement without the creatine. These findings were novel because at the time no other research had noted such improvements in body composition at the cellular and sub cellular level in resistance trained participants supplementing with creatine. The amount of creatine consumed in the study by Cribb et al was greater than the amount typically reported in previous studies (a loading dose of around 20 g/d followed by a maintenance dose of 3-5 g/d is generally equivalent to approximately 0.3 g/kg/d and 0.03 g/kg/d respectively) and the length of the supplementation period or absence of resistance exercise may explain the observed transcriptional level changes that were absent in previous studies [30,31].

Deldicque et al [32] found a 250%, 45% and 70% increase for collagen mRNA, glucose transporter 4 (GLUT4) and Myosin heavy chain IIA, respectively after 5 days creatine loading protocol (21 g/d). The authors speculated that creatine in addition to a single bout of resistance training can favor an anabolic environment by inducing changes in gene expression after only 5 days of supplementation.

When creatine supplementation is combined with heavy resistance training, muscle insulin like growth factor (IGF-1) concentration has been shown to increase. Burke et al [2] examined the effects of an 8 week heavy resistance training protocol combined with a 7 day creatine loading protocol (0.25 g/d/kg lean body mass) followed by a 49 day maintenance phase (0.06 g/kg lean mass) in a group of vegetarian and non-vegetarian, novice, resistance trained men and women. Compared to placebo, creatine groups produced greater increments in IGF-1 (78% Vs 55%) and body mass (2.2 Vs 0.6 kg). Additionally, vegetarians within the supplemented group had the largest increase of lean mass compared to non vegetarian (2.4 and 1.9 kg respectively). Changes in lean mass were positively correlated to the modifications in intramuscular total creatine stores which were also correlated with the modified levels of intramuscular IGF-1. The authors suggested that the rise in muscle IGF-1 content in the creatine group could be due to the higher metabolic demand created by a more intensely performed training session. These amplifying effects could be caused by the increased total creatine store in working muscles. Even though vegetarians had a greater increase in high energy phosphate content, the IGF-1 levels were similar to the amount observed in the non vegetarian groups. These findings do not support the observed correlation pattern by which a low essential amino acid content of a typical vegetarian diet should reduce IGF-1 production [33]. According to authors opinions it is possible that the addition of creatine and subsequent increase in total creatine and phosphocreatine storage might have directly or indirectly stimulated production of muscle IGF-I and muscle protein synthesis, leading to an increased muscle hypertrophy [2].

## Effects of creatine supplementation on predominantly aerobic exercise

Although creatine supplementation has been shown to be more effective on predominantly anaerobic intermittent exercise, there is some evidence of its positive effects on endurance activities. Branch [28] highlights that endurance activities lasting more than 150s rely on oxidative phosphorylation as primary energy system supplier. From this meta analysis [28], it would appear that the ergogenic potential for creatine supplementation on predominantly aerobic endurance exercise diminishes as the duration of the activity increases over 150s. However it is suggested that creatine supplementation may cause a change in substrate utilization during aerobic activity possibly leading to an increase in steady state endurance performance.

Chwalbinska-Monteta [34] observed a significant decrease in blood lactate accumulation when exercising at lower intensities as well as an increase in lactate threshold in elite male endurance rowers after consuming a short loading (5 days 20 g/d) CM protocol. However, the effects of creatine supplementation on endurance performance have been questioned by some studies. Graef et al [35] examined the effects of four weeks of creatine citrate supplementation and high-intensity interval training on cardio respiratory fitness. A greater increase of the ventilatory threshold was observed in the creatine group respect to placebo; however, oxygen consumption showed no significant differences between the groups. The total work presented no interaction and no main effect for time for any of the groups. Thompson et al [36] reported no effects of a 6 week 2 g CM/d in aerobic and anaerobic endurance performance in female swimmers. In addition, of the concern related to the dosage used in these studies, it could be possible that the potential benefits of creatine supplementation on endurance performance were more related to effects of anaerobic threshold localization.

## Effects of creatine supplementation on glycogen stores

It is suggested [16,37] that another mechanism for the effect of creatine could be enhanced muscle glycogen accumulation and GLUT4 expression, when creatine supplementation is combined with a glycogen depleting exercise. Whereas it has been observed [38] that creatine supplementation alone does not enhance muscle glycogen storage. Hickner et al [15] observed positive effects of creatine supplementation for enhancing initial and maintaining a higher level of muscle glycogen during 2 hours of cycling. In general, it is accepted that glycogen depleting exercises, such as high intensity or long duration exercise should combine high carbohydrate diets with creatine supplementation to achieve heightened muscle glycogen stores [39].

## Effects of creatine ingestion to improve recovery from injury, muscle damage and oxidative stress induced by exercise

Creatine supplementation may also be of benefit to injured athletes. Op’t Eijnde et al [39] noted that the expected decline in GLUT4 content after being observed during a immobilization period can be offset by a common loading creatine (20g/d) supplementation protocol. In addition, combining CM 15g/d for 3 weeks following 5 g/d for the following 7 weeks positively enhances GLUT4 content, glycogen, and total muscle creatine storage [39].

Bassit et al [40] observed a decrease in several markers of muscle damage (creatine kinase, lactate dehydrogenase, aldolase, glutamic oxaloacetic acid transaminase and glutamic pyruvic acid transaminase) in 4 athletes after an iron man competition who supplemented with 20 g/d plus 50 g maltodextrin during a 5 d period prior to the competition.

Cooke et al [41] observed positive effects of a prior (0.3 g/d kg BW) loading and a post maintenance protocol (0.1 g/d kg BW) to attenuate the loss of strength and muscle damage after an acute supramaximal (3 set x 10 rep with 120% 1RM) eccentric resistance training session in young males. The authors speculate that creatine ingestion prior to exercise may enhance calcium buffering capacity of the muscle and reduce calcium-activated proteases which in turn minimize sarcolemma and further influxes of calcium into the muscle. In addition creatine ingestion post exercise would enhance regenerative responses, favoring a more anabolic environment to avoid severe muscle damage and improve the recovery process. In addition, in vitro studies have demonstrated the antioxidant effects of creatine to remove superoxide anion radicals and peroxinitrite radicals [42]. This antioxidant effect of creatine has been associated with the presence of Arginine in its molecule. Arginine is also a substrate for nitric oxide synthesis and can increase the production of nitric oxide which has higher vasodilatation properties, and acts as a free radical that modulates metabolism, contractibility and glucose uptake in skeletal muscle. Other amino acids contained in the creatine molecule such as glycine and methinine may be especially susceptible to free radical oxidation because of sulfhydryl groups [42]. A more recent in vitro study showed that creatine exerts direct antioxidant activity via a scavenging mechanism in oxidatively injured cultured mammalian cells [43]. In a recent in vivo study Rhaini et al [44] showed a positive effect of 7 days of creatine supplementation (4 x 5 g CM 20 g total) on 27 recreational resistance trained males to attenuate the oxidation of DNA and lipid peroxidation after a strenuous resistance training protocol.

Collectively the above investigations indicate that creatine supplementation can be an effective strategy to maintain total creatine pool during a rehabilitation period after injury as well as to attenuate muscle damage induced by a prolonged endurance training session. In addition, it seems that creatine can act as an effective antioxidant agent after more intense resistance training sessions.

## Effects of creatine supplementation on range of motion

Sculthorpe et al (2010) has shown that a 5 day (25g/d) loading protocol of creatine supplementation followed by a further 3 days of 5 g/d negatively influence both active ankle dorsiflexion and shoulder abduction and extension range of movement (ROM) in young men. There are two possible theories to explain these effects: 1) Creatine supplementation increases intracellular water content resulting in increased muscle stiffness and resistance to stretch; 2) Neural outflow from the muscle spindles is affected due to an increased volume of the muscle cell. The authors highlight that the active ROM measures were taken immediately after the loading phase and the reduced active ROM may not be seen after several weeks of maintenance phase [45]. Hile et al [46] observed an increase in compartment pressure in the anterior compartment of the lower leg, which may also have been responsible for a reduced active ROM.

## Documented effects of creatine supplementation for health and clinical setting

Neurological and cognitive function has also been shown to be improved by creatine supplementation [47,48]. Rawson and Venezia [49] review the effects of creatine supplementation on cognitive function highlighting that higher brain creatine has been associated with improved neuropsychological performance. Creatine supplementation protocols have been shown to increase brain creatine and phosphocreatine contents. Cognitive processing hindered due to sleep deprivation and natural impairment due to aging can be improved by creatine supplementation. This review also highlights other possible benefits of creatine ingestion to older adults, such as improvements in: fatigue resistance, strength, muscle mass, bone mineral density, and performance of activities of daily living. Some of these benefits occur without concurrent exercise. The authors inform that discrepancies between studies do exist and are hard to explain but may be possibly due to differences in diet, race and/or supplementation protocols. However, the ideal dose of creatine to maximize brain uptake is not known. Patients have been supplemented with 40 g while in healthy adults positive results have been reported with around 20 g per day [49].

Studies with animal and cellular models demonstrated positive effect of creatine ingestion on neurodegenerative diseases. These effects have been attributed to improved overall cellular bioenergetics due to an expansion of the phosphocreatine pool [50]. Creatine deficiency syndromes, due to deficiency of glycine amidinotransferase and guanidinoacetate methyltransferase, can cause decreases or complete absence of creatine in the central nervous system. Syndromes of this nature have the possibility to be improved by supplementing orally with creatine. Brain creatine deficiency resulting from ineffective crea T1 has been shown not to be effectively treated with oral creatine supplementation [51]. Additionally, oral creatine administration in patients with myopathies has shown conflicting results depending on the type of myopathy and creatine transport systems disorders [4].

## Creatine use in children and adolescents

Creatine supplementation in the under 18 population has not received a great deal of attention, especially in regards to sports/exercise performance. Despite this, creatine is being supplemented in young, <18 years old, athletes [52,53]. In a 2001 report [52] conducted on pupils from middle and high school (aged 10 – 18) in Westchester County (USA) 62 of the 1103 pupils surveyed were using creatine. The authors found this concerning for 2 main reasons: firstly, the safety of creatine supplementation is not established for this age group and is therefore not recommended. Secondly, it was speculated that taking creatine would lead on to more dangerous performance enhancing products such as anabolic steroids. It is important to point out that this potential escalation is speculation. Furthermore, a questionnaire was used to determine creatine use amongst this age group and does not necessarily reflect the truth.

A child’s ability to regenerate high energy phosphates during high intensity exercise is less than that of an adult. Due to this, creatine supplementation may benefit the rate and use of creatine phosphate and ATP rephosporylation. However, performance in short duration high-intensity exercise can be improved through training therefore supplementation may not be necessary [54].

Based on the limited data on performance and safety, some authors have not identified any conclusions and do not recommend its consumption in regards to creatine supplementation in children and adolescents [52,54]. Conversely, according to the view of the ISSN [5], younger athletes should consider a creatine supplement under certain conditions: puberty is past and he/she is involved in serious competitive training; the athlete is eating a well-balanced caloric adequate diet; he/she as well as the parents approve and understand the truth concerning the effects of creatine supplementation; supplement protocols are supervised by qualified professionals; recommended doses must not be exceeded; quality supplements are administered.

Within this framework, creatine supplementation in young, post puberty athletes can be considered a high quality type of “food” that can offer additional benefits to optimise training outcomes.

## Dosing protocols applied in creatine supplementation

A typical creatine supplementation protocol consists of a loading phase of 20 g CM/d or 0.3 g CM/kg/d split into 4 daily intakes of 5 g each, followed by a maintenance phase of 3-5 g CM/d or 0.03 g CM/kg/d for the duration of the supplementation period [5]. Other supplementation protocols are also used such as a daily single dose of around 3 – 6 g or between 0.03 to 0.1 g/kg/d [15,55] however this method takes longer (between 21 to 28 days) to produce ergogenic effects [5]. Sale et al [56] found that a moderate protocol consisting of 20 g CM taken in 1g doses (evenly ingested at 30-min intervals) for 5 days resulted in reduced urinary creatine and methylamine excretion, leading to an estimated increase in whole body retention of creatine (+13%) when compared with a typical loading supplementation protocol of 4 x 5 g/d during 5 days (evenly ingested at 3 hour intervals). This enhancement in creatine retention would lead to a significantly higher weight gain when people follow a moderate protocol ingestion of several doses of small amounts of CM evenly spread along the day.

## Responders vs. non-responders

Syrotuik and Bell [57] investigated the physical characteristics of responder and non-responder subjects to creatine supplementation in recreationally resistance trained men with no history of CM usage. The supplement group was asked to ingest a loading dosage of 0.3 g/kg/d for 5 days. The physiological characteristics of responders were classified using Greenhaff et al [58] criterion of >20 mmol/kg dry weight increase in total intramuscular creatine and phosphocreatine and non responders as <10 mmol/kg dry weight increase, a third group labeled quasi responders were also used to classify participants who fell in between the previously mentioned groups (10-20 mmol/kg dry weight). Overall, the supplemented group showed a mean increase in total resting muscle creatine and phosphocreatine of 14.5% (from 111.12 ± 8.87 mmol/kg dry weight to 127.30 ± 9.69 mmol/kg dry weight) whilst the placebo group remained relatively unaffected (from 115.70 ± 14.99 mmol/kg dry weight to 111.74 ± 12.95 mmol/kg dry weight). However when looking at individual cases from the creatine group the results showed a variance in response. From the 11 males in the supplemented group, 3 participants were responders (mean increase of 29.5 mmol/kg dry weight or 27%), 5 quasi responders (mean increase of 14.9 mmol/kg dry weight or 13.6%) and 3 non-responders (mean increase of 5.1 mmol/kg dry weight or 4.8%). Using muscle biopsies of the vastus lateralis, a descending trend for groups and mean percentage fiber type was observed. Responders showed the greatest percentage of type II fibers followed by quasi responders and non-responders. The responder and quasi responder groups had an initial larger cross sectional area for type I, type IIa and type IIx fibers. The responder group also had the greatest mean increase in the cross sectional area of all the muscle fiber types measured (type I, type IIa and type IIx increased 320, 971 and 840 μm^2^ respectively) and non-responders the least (type I, type IIa and type IIx increased 60, 46 and 78 μm^2^ respectively). There was evidence of a descending trend for responders to have the highest percentage of type II fibers; furthermore, responders and quasi responders possessed the largest initial cross sectional area of type I, IIa and IIx fibers. Responders were seen to have the lowest initial levels of creatine and phosphocreatine. This has also been observed in a previous study [17] which found that subjects whose creatine levels were around 150 mmol/Kg dry mass did not have any increments in their creatine saturation due to creatine supplementation, neither did they experience any increases of creatine uptake, phosphocreatine resynthesis and performance. This would indicate a limit maximum size of the creatine pool.

In summary responders are those individuals with a lower initial level of total muscle creatine content, greater population of type II fibers and possess higher potential to improve performance in response to creatine supplementation.

## Commercially available forms of creatine

There are several different available forms of creatine: creatine anhydrous which is creatine with the water molecule removed in order to increase the concentration of creatine to a greater amount than that found in CM. Creatine has been manufactured in salt form: creatine pyruvate, creatine citrate, creatine malate, creatine phosphate, magnesium creatine, creatine oroate, Kre Alkalyn (creatine with baking soda). Creatine can also be manufactured in an ester form. Creatine ethyl ester (hydrochloride) is an example of this, as is creatine gluconate which is creatine bound to glucose. Another form is creatine effervescent which is creatine citrate or CM with citric acid and bicarbonate. The citric acid and bicarbonate react to produce an effervescent effect. When mixed with water the creatine separates from its carrier leaving a neutrally charged creatine, allowing it to dissolve to a higher degree in water. Manufacturers claim that creatine effervescent has a longer and more stable life in solution. When di-creatine citrate effervescent was studied [59] for stability in solution it was found that the di-creatine citrate dissociates to citric acid and creatine in aqueous solutions which in turn forms CM and eventually crystallises out of the solution due to its low solubility. Some of the creatine may also convert to creatinine.

Jager et al [60] observed 1.17 and 1.29 greater peak plasma creatine concentration 1 hour after ingesting creatine pyruvate compared to isomolar amount of CM and creatine citrate respectively. However time to peak concentration, and velocity constants of absorption and elimination, was the same for all three forms of creatine. Although not measured in this study it is questionable that these small differences in plasma creatine concentrations would have any effect on the increase of muscle creatine uptake. Jäger et al [61] investigated the effects of 28-days of creatine pyruvate and citrate supplementation on endurance capacity and power measured during an intermittent handgrip (15 s effort per 45s rest) exercise in healthy young athletes. The authors used a daily dose protocol with the intention to slowly saturate muscle creatine stores. Both forms of creatine showed slightly different effects on plasma creatine absorption and kinetics. The two creatine salts significantly increased mean power but only pyruvate forms showed significant effects for increasing force and attenuating fatigability during all intervals. These effects can be attributed to an enhanced contraction and relaxation velocity as well as a higher blood flow and muscle oxygen uptake. On the other hand, the power performance measured with the citrate forms decreases with time and improvements were not significant during the later intervals. In spite of these positive trends further research is required about the effects of these forms of creatine as there is little or no evidence for their safety and efficacy. Furthermore the regularity status of the novel forms of creatine vary from country to country and are often found to be unclear when compared to that of CM [62].

In summary, creatine salts have been show to be less stable than CM. However the addition of carbohydrates could increase their stability [62]. The potential advantages of creatine salts over CM include enhanced aqueous solubility and bioavailability which would reduce their possible gastrointestinal adverse effects [63]. The possibility for new additional formulation such as tablets or capsules is interesting for its therapeutic application due to its attributed better dissolution kinetics and oral absorption compared to CM [63]. However more complete in vivo pharmaceutical analysis of creatine salts are required to fully elucidate their potential advantages/disadvantages over the currently available supplement formulations.

Creatine is a hydrophilic polar molecule that consists of a negatively charged carboxyl group and a positively charged functional group [64]. The hydrophilic nature of creatine limits its bioavailability [65]. In an attempt to increase creatines bioavailability creatine has been esterified to reduce the hydrophilicity; this product is known as creatine ethyl ester. Manufacturers of creatine ethyl ester promote their product as being able to by-pass the creatine transporter due to improved sarcolemmal permeability toward creatine [65]. Spillane et al [65] analyzed the effects of a 5 days loading protocol (0.30 g/kg lean mass) followed by a 42 days maintenance phase (0.075 g/kg lean mass) of CM or ethyl ester both combined with a resistance training program in 30 novice males with no previous resistance training experience. The results of this study [65] showed that ethyl ester was not as effective as CM to enhance serum and muscle creatine stores. Furthermore creatine ethyl ester offered no additional benefit for improving body composition, muscle mass, strength, and power. This research did not support the claims of the creatine ethyl ester manufacturers.

Polyethylene glycol is a non-toxic, water-soluble polymer that is capable of enhancing the absorption of creatine and various other substances [66]. Polyethylene glycol can be bound with CM to form polyethylene glycosylated creatine. One study [67] found that 5 g/d for 28 days of polyethylene glycosylated creatine was capable of increasing 1RM bench press in 22 untrained young men but not for lower body strength or muscular power. Body weight also did not significantly change in the creatine group which may be of particular interest to athletes in weight categories that require upper body strength. Herda et al [68] analyzed the effects of 5 g of CM and two smaller doses of polyethylene glycosylated creatine (containing 1.25 g and 2.5 g of creatine) administered over 30 days on muscular strength, endurance, and power output in fifty-eight healthy men. CM produced a significantly greater improvement in mean power and body weight meanwhile both CM and polyethylene glycosylated form showed a significantly (p < 0.05) greater improvement for strength when compared with control group. These strength increases were similar even though the dose of creatine in the polyethylene glycosylated creatine groups was up to 75% less than that of CM. These results seem to indicate that the addition of polyethylene glycol could increase the absorption efficiency of creatine but further research is needed before a definitive recommendation can be reached.

## Creatine in combination with other supplements

Although creatine can be bought commercially as a standalone product it is often found in combination with other nutrients. A prime example is the combination of creatine with carbohydrate or protein and carbohydrate for augmenting creatine muscle retention [5] mediated through an insulin response from the pancreas [69]. Steenge et al [70] found that body creatine retention of 5 g CM was increased by 25% with the addition of 50 g of protein and 47 g of carbohydrate or 96 g carbohydrate when compared to a placebo treatment of 5 g carbohydrate. The addition of 10g of creatine to 75 g of dextrose, 2 g of taurine, vitamins and minerals, induced a change in cellular osmolarity which in addition to the expected increase in body mass, seems to produce an up regulation of large scale gene expression (mRNA content of genes and protein content of kinases involved in osmosensing and signal transduction, cytoskeleton remodelling, protein and glycogen synthesis regulation, satellite cell proliferation and differentiation, DNA replication and repair, RNA transcription control, and cell survival) [25]. Similar findings have also been reported for creatine monohydrate supplementation alone when combined with resistance training [71].

A commercially available pre-workout formula comprised of 2.05 g of caffeine, taurine and glucuronolactone, 7.9 g of L-leucine, L-valine, L-arginine and L-glutamine, 5 g of di-creatine citrate and 2.5 g of β-alanine mixed with 500 ml of water taken 10 minutes prior to exercise has been shown to enhance time to exhaustion during moderate intensity endurance exercise and to increase feelings of focus, energy and reduce subjective feelings of fatigue before and during endurance exercise due to a synergistic effect of the before mentioned ingredients [72]. The role of creatine in this formulation is to provide a neuroprotective function by enhancing the energy metabolism in the brain tissue, promoting antioxidant activities, improving cerebral vasculation and protecting the brain from hyperosmotic shock by acting as a brain cell osmolyte. Creatine can provide other neuroprotective benefits through stabilisation of mitochondrial membranes, stimulation of glutamate uptake into synaptic vesicles and balance of intracellular calcium homeostasis [72].

## Safety and side effects of creatine supplementation

There have been a few reported renal health disorders associated with creatine supplementation [73,74]. These are isolated reports in which recommended dosages are not followed or there is a history of previous health complaints, such as renal disease or those taking nephrotoxic medication aggravated by creatine supplementation [73]. Specific studies into creatine supplementation, renal function and/or safety conclude that although creatine does slightly raise creatinine levels there is no progressive effect to cause negative consequences to renal function and health in already healthy individuals when proper dosage recommendations are followed [73-77]. Urinary methylamine and formaldehyde have been shown to increase due to creatine supplementation of 20 g/d; this however did not bring the production outside of normal healthy range and did not impact on kidney function [56,78]. It has been advised that further research be carried out into the effects of creatine supplementation and health in the elderly and adolescent [73,75]. More recently, a randomized, double blind, 6 month resistance exercise and supplementation intervention [79] was performed on elderly men and women (age >65 years) in which subjects were assigned to either a supplement or placebo group. The supplement group was given 5 g CM, 2 g dextrose and 6 g conjugated linoleic acid/d, whilst the placebo group consumed 7 g dextrose and 6 g safflower oil/d. CM administration showed significantly greater effects to improve muscular endurance, isokinetic knee extension strength, fat free mass and to reduce fat mass compared to placebo. Furthermore the supplement group had an increase in serum creatinine but not creatinine clearance suggesting no negative effect on renal function.

Cornelissen et al [80] analyzed the effects of 1 week loading protocol (3 X 5 g/d CM) followed by a 3 month maintenance period (5 g/d) on cardiac patients involved in an endurance and resistance training program. Although CM supplementation did not significantly enhance performance, markers of renal and liver function were within normal ranges indicating the safety of the applied creatine supplementation protocol.

A retrospective study [81], that examined the effects of long lasting (0.8 to 4 years) CM supplementation on health markers and prescribed training benefits, suggested that there is no negative health effects (including muscle cramp or injuries) caused by long term CM consumption. In addition, despite many anecdotal claims, it appears that creatine supplementation would have positive influences on muscle cramps and dehydration [82]. Creatine was found to increase total body water possibly by decreasing the risk of dehydration, reducing sweat rate, lowering core body temperature and exercising heart rate. Furthermore, creatine supplementation does not increase symptoms nor negatively affect hydration or thermoregulation status of athletes exercising in the heat [83,84]. Additionally, CM ingestion has been shown to reduce the rate of perceived exertion when training in the heat [85].

It is prudent to note that creatine supplementation has been shown to reduce the body’s endogenous production of creatine, however levels return to normal after a brief period of time when supplementation ceases [1,6]. Despite this creatine supplementation has not been studied/supplemented with for a relatively long period. Due to this, long term effects are unknown, therefore safety cannot be guaranteed. Whilst the long term effects of creatine supplementation remain unclear, no definitive certainty of either a negative or a positive effect upon the body has been determined for many health professionals and national agencies [19,78]. For example the French Sanitary Agency has banned the buying of creatine due to the unproven allegation that a potential effect of creatine supplementation could be that of mutagenicity and carcinogenicity from the production of heterocyclic amines [78]. Long term and epidemiological data should continue to be produced and collected to determine the safety of creatine in all healthy individuals under all conditions [78].

## Conclusion and practical recommendations

The above review indicates that creatine supplementation has positive effects on:

· Amplifying the effects of resistance training for enhancing strength and hypertrophy [5,22,28].

· Improving the quality and benefits of high intensity intermittent speed training [21].

· Improving aerobic endurance performance in trials lasting more than 150s [7].

· Seems to produce positive effects on strength, power, fat free mass, daily living performance and neurological function in young and older people [49].

· Research on the mechanisms of creatines effect has progressed since 2007 showing an up regulation of gene expression when creatine is administered together with resistance training exercises.

· Regarding predominantly aerobic endurance performance, the increased bodies’ creatine stores, seems to amplify favorable physiological adaptations such as: increased plasma volume, glycogen storage, improvements of ventilatory threshold and a possible reduction of oxygen consumption in sub maximal exercise.

A typical creatine supplementation protocol of either a loading phase of 20 to 25 g CM/d or 0.3 g CM/kg/d split into 4 to 5 daily intakes of 5 g each have been recommended to quickly saturate creatine stores in the skeletal muscle. However a more moderate protocol where several smaller doses of creatine are ingested along the day (20 intakes of 1 g every 30 min) could be a better approach to get a maximal saturation of the intramuscular creatine store. In order to keep the maximal saturation of body creatine, the loading phase must be followed by a maintenance period of 3-5 g CM/d or 0.03 g CM/kg/d. These strategies appear to be the most efficient way of saturating the muscles and benefitting from CM supplementation. However more recent research has shown CM supplementation at doses of 0.1 g/kg body weight combined with resistance training improves training adaptations at a cellular and sub-cellular level. Creatine retention by the body from supplementation appears to be promoted by about 25% from the simultaneous ingestion of carbohydrate and/or protein mediated through an increase in insulin secretion. This combination would produce a faster saturation rate but has not been shown to have a greater effect on performance.

Different forms of creatine in combination with other sports supplements as well as varying doses and supplementation methodology should continue to be researched in an attempt to understand further application of creatine to increase sports and exercise performance of varying disciplines. It is important to remain impartial when evaluating the safety of creatine ingested as a natural supplement. The available evidence indicates that creatine consumption is safe. This perception of safety cannot be guaranteed especially that of the long term safety of creatine supplementation and the various forms of creatine which are administered to different populations (athletes, sedentary, patient, active, young or elderly) throughout the globe.

## Abbreviations

ATP, Adenosine triphosphate; CM, Creatine monohydrate; ES, Effect size; g/d, Grams per day; g/kg/d, Grams per kilogram of body mass per day; ROM, Range of movement.

## Competing interests

Maxinutrition and the University of Greenwich are providing joint funding with to one of the author’s PhD project; however, this does not affect the purpose of the review and its content.

## Authors’ contributions

All authors have read, reviewed and contributed to the final manuscript.
